# Logical Inference Framework for Experimental Design of Mechanical Characterization Procedures

**DOI:** 10.3390/s18092984

**Published:** 2018-09-07

**Authors:** Guillermo Rus, Juan Melchor

**Affiliations:** 1 Department of Structural Mechanics, University of Granada, 18071 Granada, Spain; grus@ugr.es; 2 Biosanitary Research Institute, 18016 Granada, Spain; 3 MNat Scientific Unit of Excellence, University of Granada, 18071 Granada, Spain

**Keywords:** inverse problem, inference Bayesian updating, model-class selection, stochastic inverse problem, probability logic, experimental design

## Abstract

Optimizing an experimental design is a complex task when a model is required for indirect reconstruction of physical parameters from the sensor readings. In this work, a formulation is proposed to unify the probabilistic reconstruction of mechanical parameters and an optimization problem. An information-theoretic framework combined with a new metric of information density is formulated providing several comparative advantages: (i) a straightforward way to extend the formulation to incorporate additional concurrent models, as well as new unknowns such as experimental design parameters in a probabilistic way; (ii) the model causality required by Bayes’ theorem is overridden, allowing generalization of contingent models; and (iii) a simpler formulation that avoids the characteristic complex denominator of Bayes’ theorem when reconstructing model parameters. The first step allows the solving of multiple-model reconstructions. Further extensions could be easily extracted, such as robust model reconstruction, or adding alternative dimensions to the problem to accommodate future needs.

## 1. Introduction

Inverse problems are used in various fields, including medical imaging, nondestructive testing, mathematical finance, astronomy, geophysics, or sub-surface prospecting, whenever interrogating phenomena or properties of a system that cannot be readily quantified. The inverse problem can be defined in opposition to the forward problem. Given a physical system, the forward problem consists of using an idealized model of that system to predict the outcome of possible experiments. In contrast, the inverse problem is posed to interrogate or reconstruct an unknown part of the system given an observed set of output data.

This reconstruction problem was historically first solved in a deterministic way, providing a unique answer to the unknown parameters [1,2,3]. However, if the degree of certainty and reliability of the parameters is relevant, a probabilistic approach is required. This was introduced using the framework of Bayesian statistics by Cox and Jaynes [4] based on Cox’s postulates [5], and still being developed [6,7,8,9,10,11,12,13]. Its central idea is that the unknown is defined as a probability density function over the model parameters to be reconstructed, and this probability is updated with the experimental information and linked with a model through Bayes’ theorem. An alternative theoretical framework was posed by Tarantola [14] based on the idea of conjunction of states of information (theoretical, experimental, and prior information, generally on model parameters). The axioms of probability theory apply to different situations: the Bayesian perspective is the traditional statistical analysis of random phenomena, whereas the information states criterion is the description of (more or less) subjective states of information on a system. However, the collection of applications successfully solved by Ensemble Kalman Filter-type algorithms (EnKF) are not directly solvable by the proposed formulation, at least without profound adaptations.

Furthermore, its delicate formulation poses difficulties when modifying and extending it to solve real-world needs. To overcome this, we propose an information-theoretic reconstruction framework, which is built on a new metric of information density that drops Cox’s normalization in favor of simplifications. This metric is used with the concept of combining information density functions from two independent sources: (i) experimental measurements and (ii) mathematical models, over the same data (observations and model parameters) with the aim of finding which ones are all plausible at the same time. This new framework ultimately allows the straightforward solving of problems combining multiple concurrent models, or conveniently solve as experimental design, sensor design and placement problems in specific cases as in the ultrasonics testing in a probabilistic way, which only recently has been computed from the Bayesian perspective [15,16,17,18]. Beyond this, new dimensions can be defined into the problem to accommodate future needs. Moreover, models are not required to be causal, paving the way to contingent models such as stochastic associations, for instance, whose scope extends to applications such as image reconstruction, face recognition or complex physics-based model parameters reconstruction.

**Figure 1:**
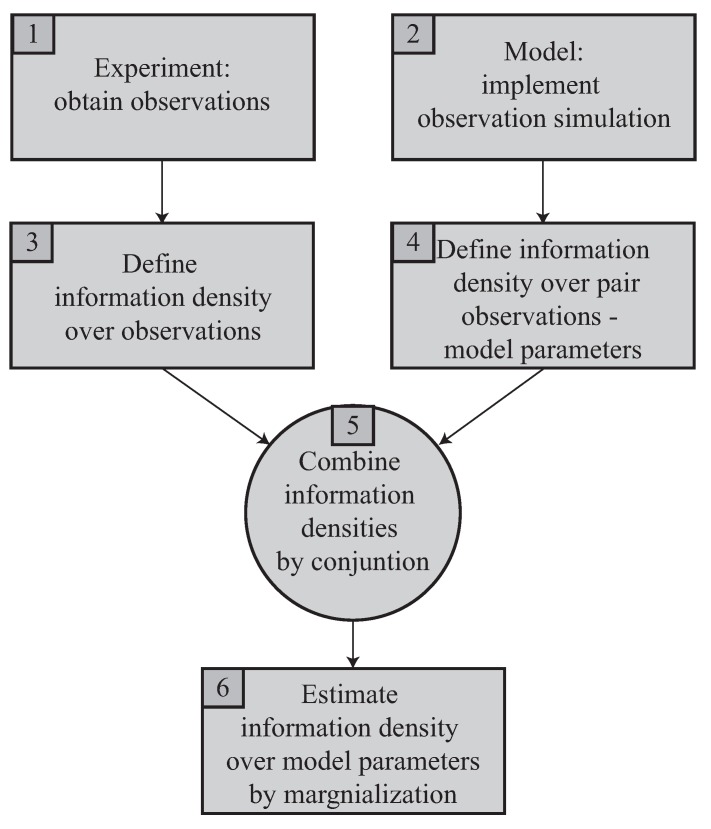
Conceptual framework of the basic information-theoretic probabilistic inverse problem.

The next section details and formalizes the procedure to solve the problem, whose components are reorganized and outlined in Figure 1.

As follows. The two mentioned starting ingredients at the top are an experiment performed to capture some measurements, in box 1, and, in box 2, an idealization of the experimental system made throughout a model, which allows simulation of the measurements, but depends on the model parameters, which are the unknowns of the problem. In box 3, to treat the observations from box 1 in an uncertain way, they are described by means of the concept of information density over the theoretically possible space of observations, formally defined in Section 2.1 and Section 2.2. In box 4, the pairs of values of sought model parameters and simulated observations are analogously defined by means of their joint information density. In box 5, both sources of information, experiment and model, are combined as described in Section 2.2. In box 6, the probabilistic reconstruction answer is yielded as described in Section 2.4.

This scheme solves the basic form of the reconstruction inverse problem, assuming a single model and a predetermined way of measuring. However, the formulation proposed below has the strength of being easily extended to solve practical problems explained in Section 2.3, where the former assumptions need not be made.

In this work we propose a new technique to optimize the experimental design of a testing system or sensor and illustrate it for the particular case of characterizing a viscoelastic material, step by step. First, the information-theoretic inverse problem framework is formulated, then, the practical method is detailed describing the process of parametrization, the operation with discrete observation data or signals, and two key extensions: to hypothesis testing and to experimental design optimization. The proposal is illustrated with a practical example to reconstruct a mechanical model from a tensile testing.

## 2. Theory

### 2.1. Definition of Basic Variables

Assuming that the two sources of information are the experimental observations and an idealized model that simulates observations for given model parameters, two basic variables stem from this premise: observations and model parameters.

The observations O are, in the most general case, vectors compounding a set of signals $o_{i}\left(t\right)$, but may also be a single signal, analog or digitally sampled, sets of values, or even a single measurement value. Although a unique space of observations O can be defined to contain all possible observations, depending on their origin they can be either observed $O^{o}=\left\{o_{i}^{o}\left(t\right)\right\}$ or modeled $O^{m}=\left\{o_{i}^{m}\left(t\right)\right\}$. Examples of observations may be ultrasonic or seismic recorded signals, optical, X-ray or thermographic images, or any measurement based on any physical magnitude used to interrogate the system under study.

The model parameters M are analogously a set of diverse physical parameters, which define a manifold H. They are the input of the mathematical model that simulates the experimental behavior and its measurable by an output. They may stand for damage parameters, pathology or sought mechanical properties, for instance, that feed models that simulate the observations described above. In the numerical example in Section 4, combining three sources of information is tested: model-based forecast, observation, and experimental design parameters for its optimization.

### 2.2. Definition of Information Density and Its Operations

To treat this data O and M in an uncertain way, we do not define univocal values, but information densities over them. The information density f(x) over either of them (*x* for generality) is defined from the conception of Cox [5] as a degree of belief or certainty that the values of *x* are plausible. Therefore, the probabilities that are established as a consequence of this logical framework are objective and the logical relations in that axiomatization [19,20]. They can be understood as states of knowledge, in contrast to the physical propensity of a phenomenon. A more detailed discussion is provided in [21]. The present definition of information density is compatible with either the evidential, logical and even subjective theoretical frameworks described in [21].

In particular, we formally define the information density f(x) of an event or value *x* as a nonnegative real $f\left(x\right)\in R^{+}$ that is zero (f(x)=0) when its value is impossible, and the larger the more plausible. Two logical inference operations introduce a structure to the space of all probability distributions. Starting from the *and* and *or* operator definition for Boolean logic (which can adopt the values of true or false, without intermediate degrees of certainty), over two probability distributions $P_{a}$ and $P_{b}$ that may represent two different sources of information *a* and *b* about the same events,
$P_{a}$$P_{b}$$P_{a}\;and\;P_{b}$$P_{a}\;or\;P_{b}$falsefalsefalsefalsefalsetruefalsetruetruefalsefalsetruetruetruetruetrue
the simplest logical operations over information densities *f* that fulfill these axioms are,

(1) $$\begin{array}{cc} \left\{f_{1}\;or\;f_{2}\right\} & =f_{1}+f_{2}\\ \left\{f_{1}\;and\;f_{2}\right\} & =f_{1}f_{2} \end{array}$$

Note that the normalization requirement of either Kolmogorov axioms or Cox’s postulates (Kolmogorov axioms state that the probability *P* of any events *A*, *B* satisfy [22],

1. Non-negativity: P(A)≥0.
2. Finite additivity: P(A∪B)=P(A)+P(B)∀A,B|A∩B=∅.
3. Normalization: P(Ω)=1.)

is not imposed here, which will strongly simplify the formulation, as shown later. In particular, dropping the normalization axiom in the definition of the information density *f* simplifies the final formulation in comparison with the Bayesian inverse problem as well as theory of Tarantola.

A main cornerstone of this formulation is that the relationship between the model parameters and the observations provided by a model need not to be an implication due to a cause-effect, which requires to define the conditional probability of Bayesian statistics. Instead, just the conjunction of information densities needs to be defined, in which the causality between model and observations may be inverted or even not exist, as further discussed in [21]. These two characteristics define the relationship between model and observation. One uses probability as logic, and alternative one interprets it as information content. They will be shown below to allow the solving of reconstruction problems with multiple concurrent models, also paving the way to contingent models such as stochastic associations, as well as experimental design and placement problems, in a simple and straightforward way, both conceptually and computationally.

Therefore, we define the information contents that come from the observations as $f^{o}\left(O\right)$, and that provided by the model as $f^{m}\left(O,M\right)$, in the sense that the model couples values of model parameters M with observations O, yielding degrees of certainty *f* when the fed values in the model is fulfilled or not with a range of degrees of plausibility.

The origin of the uncertainties is, therefore, incorporated into the interpretation of probability as a measure of relative plausibility of the various possibilities obtained from the available information. This interpretation is not well known in the engineering community where there is a wide-spread belief that probability only applies to aleatory uncertainty (inherent randomness in nature) and not to epistemic uncertainty (missing information). Jaynes [4] noted that the assumption of inherent randomness is an example of what he called the Mind-Projection Fallacy: our uncertainty is ascribed to an inherent property of nature, or, more generally, our models of reality are confused with reality.

The interpretation of the final inferred model probability can be used either to identify a set of plausible values, or to find the most probable one (expected, i.e., that with maximal information density, argmax f(M)), or, following Tarantola [14], just to falsify inconsistent models (those with low *f*), since according to Poppers falsationism [23], that is the only thing we can assert.

### 2.3. Definition of Extended Variables

The first extension that this framework allows is the case when several models can be combined. The model forecast may therefore also depend on the hypothesis we assume about the model physics, which in turn implies distinct sets of model parameters for each hypothesis. This brings in the hypothesis H within a set H (which is usually a discrete manifold, but not necessarily) as a new uncertain variable, which conditions the number of unknowns and therefore the model complexity. For instance, decisions can be made on whether some model parameters are known from literature or treated as unknowns to be sought. Alternatively, models can be added or removed in hierarchical combinations (for instance as multiscale models) or in parallel, as illustrated and clarified in the numerical example at the end of this work.

**Figure 2:**
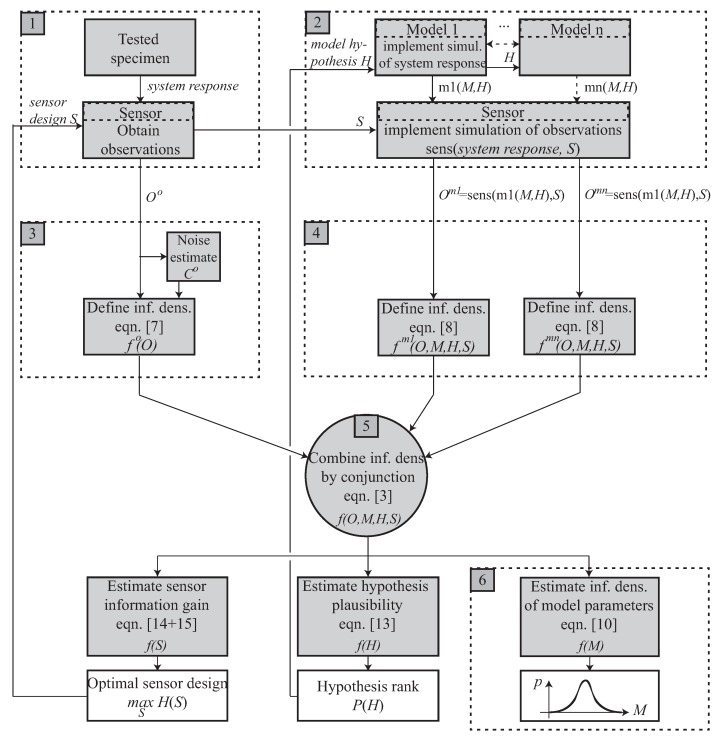
Flow chart of the complete information-theoretic probabilistic inverse problem. The abbreviation “inf. dens.” is referred to information density.

This extension to consider several hypotheses on the model or models is included in box 2 of the flow chart in Figure 2 by multiplying the possible models and making them dependent on the hypothesis H.

On the other hand, in real practice, the way the system is interrogated must be decided. This implies a problem of sensor optimization and even in experiments for large-scale [24,25,26,27], in the wide sense of sensors, either as positioning, their internal design, any measurement filtering or signal processing aimed at extracting the signal parts with most useful information while minimal noise, or the measurement domains (time, frequency, phase, cepstrum, etc.). This gives rise to a set of sensor parameters S within a manifold of possible values S, which become the variables to optimize. The sensor placement optimization procedure will be described and illustrated in detail in a future work.

This extension to consider the design of the interrogation system is included in boxes 1 and 2 of the flow chart in Figure 2 by splitting the experiment into the system and the sensor that captures its response, which depends on the experimental design parameters S.

Both H and S are analogously defined in a probabilistic sense by means of information densities defined over their spaces H and S, yielding the information contents provided by the observations as $f^{o}\left(O,S\right)$, and those provided by each concurrent model *n* as $f^{m_{n}}\left(O,M,H,S\right)$, or, in the case of a single model, just $f^{m}\left(O,M,H,S\right)$.

Analogously to the extension to H and S, further dimensions could be easily extended to the formulation, to accommodate future needs.

### 2.4. Information Theory Inverse Problem

Recall the flow chart in Figure 1 and focus on the concept of information density functions *f*, which are combined using the logical and operator [21,28,29]. Then, we have two or more sources of information (probabilistic propositions) to infer information about the model parameters M. We introduce, i) a source from experimental observations of the system $f^{o}$, and ii) a source from a mathematical model of the system $f^{m}$, in this case, the probabilistic logic conjunction operator allows computation of the information state that the system parameters fulfill both propositions simultaneously, $\left\{f^{o}\;and\;f^{m}\right\}$, as,

(2) $$\begin{array}{ccc} f(O,M,H,S) & = & \left\{f^{o}\left(O,M,H,S\right)\;and\;f^{m}\left(O,M,H,S\right)\right\}\\ & = & f^{o}\left(O,M,H,S\right)f^{m}\left(O,M,H,S\right) \end{array}$$

Note that the simultaneity of the propositions relieves the causality requirement of the Bayesian framework. Following the basics of the scientific method for physical sciences, hypotheses are proposed that explain the observations, which, in our case, are materialized as models that try to be predictive. The next step is the hypothesis validation by confronting those predictions against the observations, which is here formulated with the aim of partial degrees of certainty as the conjunction of certainty of predictions being true at the same time as observations. This is parallel and therefore consistent with hypothesis testing.

In addition, multiple models can be combined, following Figure 2. If several models ($m_{1}$, $m_{2}$, …) provide information, the joint information can be generalized as,

(3) $$\begin{array}{ccc} f(O,M,H,S) & = & \left\{f^{o}\;and\;f^{m_{1}}\;and\;f^{m_{2}}\;and\;\ldots \right\}\\ & = & f^{o}\left(O,M,H,S\right)f^{m_{1}}\left(O,M,H,S\right)f^{m_{2}}\left(O,M,H,S\right)\ldots \end{array}$$

Note that these models may possibly relate different subsets of model parameters, or just represent competing hypothesized imperfect models relating the same parameters in an attempt to make robust predictions in the case none of the available models perfectly predict observations.

Assuming that the experimental information on observations is carried out with sensors that are independent of techniques to infer experimental information on model parameters, and the same is true for model hypothesis and experimental designs, the joint density can be split as the product $f^{o}\left(O,M,H,S\right)=f^{o}\left(O\right)f^{o}\left(M\right)f^{o}\left(H\right)f^{o}\left(S\right)$. Note that $f^{o}\left(M\right)=1$ is the noninformative density function or constant. This is not true for the model information $f^{m}$, since it relates observations and model. In the case of the observational world, as opposed to the simulations, superscripted by *m*, $f^{o}\left(S\right)$ which usually is no information (noninformative uniform distribution). However, depending on the experimental design, the observations may be of different size or even nature (for instance measuring at different points or even measuring velocities instead of displacements, for instance), which makes $f^{o}\left(O\right)$ dependent on *S* in the sense that the structure of *O* changes, but not that the information density on *S* modifies the information density on *O*.

The reconstructed probability for the model parameters M providing the model hypothesis $H_{j}$ and experimental design $S_{l}$ is obtained from the joint probability f(O,M,H,S) in Equation (3) by extracting the marginal probability for all possible observations O∈O and provided the model hypothesis $H_{k}\in H$ is assumed to be true ($f^{o}\left(H=H_{k}\right)=1$) and one experimental design $S_{l}\in S$ as,

(4) $$\begin{array}{ccc} f\left(M\right){|}_{H=H_{k},S=S_{l}} & = & k_{1}\int_{O}f^{o}\left(O\right)f^{o}\left(M\right)f^{m}\left(O,M,H_{k},S_{l}\right)dO \end{array}$$

where $k_{1}$ is a normalization constant that replaces the dropped model hypothesis probability, which can be removed since *f* is unscaled. Note that here, ’marginal probability’ is defined in the loose sense of dropping the scaling. The assumption of no prior knowledge about the model parameters is represented by the noninformative distribution, i.e., an arbitrary constant in the assumed case of Jeffrey’s parameters $f^{o}\left(M\right)=1$, leaving,

(5) $$f\left(M\right){|}_{H=H_{k},S=S_{l}}=\int_{O}f^{o}\left(O\right)f^{m}\left(O,M,H_{k},S_{l}\right)dO$$

## 3. Method

### 3.1. Model Parametrization

The mathematical model of the experimental system maps a set of model parameters M to simulated observations O, following some idealizations, in turn based on some hypothesis H and interrogation system design S. Note that this mapping can range from a cause-effect physical relationship to just a contingent stochastic association.

The present inverse problem formulation requires that the model parameters are of Jeffrey’s type, which have the characteristic of being positive and as popular as their inverses [14]. If parameters are of Jeffrey’s type, the present formulation can be shown to be equivalent to the Bayesian framework except for a constant, which is detailed later in Section 3.6. The benefits are that all noninformative densities are constant and therefore dropped from the formulation. This assumption is required for the definition above of the logical operators and and or, as well as for defining noninformative densities as constants $f^{o}=1$ to be fully correct.

Many model parameters are non-Jeffreys, which is evident in the following example. If two materials with different elasticities (for instance of twice Young’s modulus) are compared in terms of their stiffness and compliance (its inverse), different distances are obtained. Since there is no reason to prefer one over the other, the definition of their distance should be independent of the choice, which can be attained through a logarithmic change of variable [30,31]. This change of variables is completed with a mapping from ${\tilde{m}}_{i}\in \left[0,1\right]$ to a predefined range of physically reasonable values $m_{i}\in \left[m_{i}^{\inf},m_{i}^{\sup}\right]$, to improve numerical stability, as,

(6) $${\tilde{m}}_{i}=\frac{ln\left(\frac{m_{i}}{m_{i}^{\inf}}\right)}{ln\left(\frac{m_{i}^{\sup}}{m_{i}^{\inf}}\right)}\;m_{i}=m_{i}^{\inf}e^{{\tilde{m}}_{i}ln\left(\frac{m_{i}^{\sup}}{m_{i}^{\inf}}\right)}$$

### 3.2. Particularization for Set of Discrete Observations with Gaussian Uncertainties

Observations are usually assumed to follow a Gaussian distribution $O∼N(E\left[O^{o}\right],C^{o})$ whose mean is that of the experimental observations $O^{o}$ and whose covariance matrix $C^{o}$ quantifies the measurement error noise [32,33,34]. Likewise, the numerical errors from model *m* may also be assumed Gaussian $O∼N(O^{m},C^{m})$ centered at the numerically computed ones $E\left[O^{m}\right]=O\left(H\right)$, where $E[O^{m}]$ is the m-dimensional mean vector of O, with covariance matrix $C^{m}$. However, the numerical errors are oftentimes negligible compared to the experimental ones. The density fulfilling both propositions $f^{o}$ and $f^{m}$ is similar with the likelihood density under Gaussian assumption with $O∼N(O^{m},C^{m}+C^{o})$.

Recall that the observations O are a discrete vector $O=\{o_{i}\}$, $i\in [1\ldots N_{i}]$, and that the assumptions made above are valid for every sample *i*. In addition, considering the compound probability of the information of the sensors and all instants of time is the product of each one individually, it supposes independence of information, and this product is equivalent to a sum within the exponential and the Gaussian distribution that allows an explicit expression of probability densities,

(7) $$\begin{array}{ccc} f^{o}\left(o_{i}\left(t\right)\right) & = & k_{2}\exp\left\{\begin{array}{c} -\frac{1}{2}\int \sum_{i,j=1}^{N_{i}}\left(o_{i}\left(t\right)-o_{i}^{o}\left(t\right)\right)\\ \;{\left(C_{ij}^{o}\right)}^{-1}\left(o_{j}\left(t\right)-o_{j}^{o}\left(t\right)\right)dt \end{array}\right\} \end{array}$$

(8) $$\begin{array}{ccc} f^{m}\left(o_{i}\left(t\right),M,H_{k},S_{l}\right) & = & k_{3}\exp\left\{\begin{array}{c} -\frac{1}{2}\int \sum_{i,j=1}^{N_{i}}\left(o_{i}\left(t\right)-o_{i}\left(t,M,H_{k},S_{l}\right)\right)\\ \;{\left(C_{ij}^{m}\right)}^{-1}\left(o_{j}\left(t\right)-o_{j}\left(t,M,H_{k},S_{l}\right)\right)dt \end{array}\right\} \end{array}$$

(9) $$\begin{array}{ccc} \Rightarrow \;f\left(M\right){|}_{H=H_{k},S=S_{l}} & = & k_{4}\exp\overset{-J(M,H_{k},S_{l})}{\overset{︷}{\left\{\begin{array}{c} -\frac{1}{2}\int \sum_{i,j=1}^{N_{i}}\left(o_{i}\left(t,M,H_{k},S_{l}\right)-o_{i}^{o}\left(t\right)\right)\\ \;{\left(C_{ij}^{o}\;+C_{ij}^{m}\right)}^{\;-1}\left(o_{j}\left(t,M,H_{k},S_{l}\right)-o_{j}^{o}\left(t\right)\right)dt \end{array}\right\}}} \end{array}$$

The term $J(M,H_{k},S_{l})$ corresponds to a misfit function between model and observations,

(10) $$f\left(M\right){|}_{H=H_{k},S=S_{l}}=k_{4}e^{-J(M,H_{k},S_{l})}$$

The mode criterion can be adopted as it finds the most probable model parameter. Finally, if classical probability densities $\hat{f}\left(M\right){|}_{H=H_{k},S=S_{l}}=k_{5}e^{-J(M,H_{k},S_{l})}$ are desired, the constant $k_{6}$ is derived by imposing the theorem of total probability since the latter is defined by normalization to 1 as,

(11) $$\int_{M}\hat{f}\left(M\right){|}_{H=H_{k},S=S_{l}}dM=1=k_{5}I,\;I=\int_{M}e^{-J(M,H_{k},S_{l})}dM\;\Rightarrow \;k_{5}=\frac{1}{I}$$

### 3.3. Extension to Model Hypothesis Selection

As introduced above, the probabilistic nature of the reconstruction is partly motivated by the fact that the model itself may not necessarily reproduce or fully explain the experimental setup. If several models (or hypothesis within the model) are candidates based on different hypothesis $H_{k}$ about the system, the previous probabilistic formulation of the inverse problem also provides information to rank them. The underlying idea is the following: if the model hypothesis is considered to be an uncertain discrete variable, its probability can eventually be extracted as a marginal probability from Equation (3). The probability of each hypothesis will therefore have the sense of degree of certainty of being true in the sense that the probabilistic conjunction of certainty (or information) provided by the experimental measurements and model are coherent [9,11].

The goal is to find the probability f(H), understood as a measure of plausibility of a model hypothesis H [13], or in other words, the information gain it provides, or how much can be learned by using the hypothesized model. It is simply derived as the marginal probability of the posterior probability f(O,M,H,S) defined in Equation (3),

(12) $$\begin{array}{ccc} f\left(H\right){|}_{S=S_{l}} & = & \int_{O}\int_{M}f\left(O,M,H,S_{l}\right)dMdO\\ & = & k_{6}f^{o}\left(H\right)\int_{O}\int_{M}f^{o}\left(O\right)f^{o}\left(M\right)f^{m}\left(O,M,H,S_{l}\right)dMdO \end{array}$$

If no prior information is provided by the user about the hypothesis then $f^{o}\left(H\right)=1$. Furthermore, this procedure involves the same integral as that for the constant $k_{5}$, i.e., allowing to reuse the integral defined in Equation (11),

(13) $$f\left(H\right){|}_{S=S_{l}}=k_{6}f^{o}\left(H\right)\int_{M}f\left(M\right){|}_{H=H_{k},S_{l}}dM=k_{6}I$$

where the normalization constant $k_{6}$ comes from grouping previous constants that multiply and it can be solved from the theorem of total probability over all hypothesis $H=\{H_{k}\}$ to obtain probabilities, $\sum_{H}p\left(H_{k}\right)=1$.

Note that multiple dimensions of the problem can be coupled to try to solve problems such as robust parameter reconstruction [35], for instance, or others defined in future needs. The procedure for robust parameter reconstruction would imply a first step where the hypothesis plausibility $f\left(H\right){|}_{S=S_{l}}$ is computed using Equation (13), followed by a second step where the model parameters plausibility f(M) is computed using an alternative derivation of Equation (5) without restricting to a particular hypothesis $H_{k}$, but rather incorporating all of them, by,

(14) $$\begin{array}{ccc} f\left(M\right){|}_{S=S_{l}} & = & \sum_{H}\int_{O}f^{o}\left(O\right)f\left(H_{k}\right){|}_{S=S_{l}}f^{m}\left(O,M,H_{k},S_{l}\right)dO \end{array}$$

Note that the space of hypothesis is discrete, so rather than integrating over it, a sum is formulated.

### 3.4. Extension to Interrogation System Design

Recall that by interrogation system design, we may understand any mapping from experimental output to recorded signals, which may range from experimental design parameters, the positioning of the sensors, any measurement filtering aimed at extracting the signal parts with most useful information while minimal noise, or the measurement domains (time, frequency, phase, cepstrum, etc.), with the same goal.

This goal is formulated as finding the S that maximizes the information density f(S). It may be more useful to understand it as maximizing the information entropy H(S)=p(S)logp(S), which is a measure of the information contents [36]. The information density is again derived as the marginal probability of the posterior probability f(O,M,H,S) defined in Equation (3), assuming no prior information about the sensors nor model parameters,

(15) $$\begin{array}{ccc} f\left(S\right){|}_{H=H_{k}} & = & \int_{O}\int_{M}f\left(O,M,H_{k},S\right)dMdO\\ & = & \int_{O}\int_{M}f^{o}\left(O\right)f^{m}\left(O,M,H_{k},S\right)dMdO \end{array}$$

and can be interpreted as the information gain or a measure of what can be learned for every value of S. Instead, the information theory community typically operates with information entropy (measured in bits, nits or hartleys depending on whether the log base is 2, *e* or 10) which is readily obtained from the probability, which in turn comes from normalizing the information density to fulfill the theorem of total probability,

(16) $$\begin{array}{c} H\left(S\right)=p\left(S\right)\log p\left(S\right)\;p\left(S\right)=k_{7}f\left(S\right)\;\left|\;\sum_{S}p\left(S\right)=1\right. \end{array}$$

If a reliability or cost of failure related criterion is preferred for the sensor optimization, the probability curve p(S) should be computed instead of the entropy, since p(S) is directly related to the reliability R=1−P(failure), whereas the cost efficiency can be attained by estimating the total probabilistic cost as the sum of the cost of sensors, that may depend on their configuration and number S, and the cost of failure, which in turn depends on p(S).

### 3.5. Summary of Extended Framework

The variables and equations described above are organized in the flow-chart in Figure 2, which details the concepts outlined in Figure 1, where the extensions are clearly marked. It starts from the ingredients at the top and yields the answers at the bottom, from left to right.

In particular, note that, beyond the standard inverse problem goal of estimating the model parameters (box 6), the framework delivers (i) the sensor information gain depending on its design, which allows optimization of the experimental design or placement, and (ii) the plausibility of alternative model hypothesis, which allows ranking and choosing among plausible mathematical idealizations of the physical system. In addition, note that multiple models may be concurrently adopted (unfolding of box 2), which provides practical solutions where multimodal, multiscale or multiphysics are relevant.

### 3.6. Validation

A simple procedure to validate the provided formulation is to compare the model reconstruction Equation (3) and the hypothesis ranking Equation (13) with those obtained using the Bayesian framework by Beck [9,37],

$$\begin{array}{ccc} p_{D}\left(M\right) & = & \frac{p\left(D|M,H\right)p_{o}\left(M|H\right)}{p(D|H)}\\ p(H_{j}|D,M) & = & \frac{p\left(D|H_{j},M\right)p\left(H_{j}|M\right)}{p(D|M)} \end{array}$$

where D stands for the data, M the model parameters, H the model class, and $p_{o}$ is the prior on the model parameters [14], respectively. Note that all formulations imply the same computations except for a computationally expensive constant, whose computation is typically omitted and adjusted using the theorem of total probability, which coincides exactly with the proposed procedure when extended to computing the posterior probability *p* by normalizing the information contents *f*. The computation of the integral is here avoided for the model parameter reconstruction, and only needed for hypothesis testing and experimental design optimization. The Bayesian concepts used in this study requires Jeffrey’s type parameter, which is obtained through a logarithmic mapping, and is used combining a priori information from two independent sources over the observations and model parameters, to find the plausibility of them simultaneously.

## 4. Example

To illustrate the utility and effectiveness of the proposed method, a simple but nontrivial inverse problem is solved. It consists of a tensile test where the sensor is a generic displacement sensor characterized by its measurement error. The requested results are the constitutive nonlinear viscoelastic mechanical constants of a quasi-incompressible soft tissue sample. However, the extended formulation allows the easy ranking of the plausibility of several models detailed below, as well as optimizing the interrogation system, also detailed later.

The following constitutive laws are hypothesized:

$H_{1}$: Maxwell viscoelasticity that additively combines strains from damper of viscosity η and nonlinear elastic spring described by shear modulus μ and nonlinearity of first order Landau-type A, which relates shear stress σ and strain ε, with parameters constants M={μ,η,A} being the output of the model governed by,
  (17) $$\varepsilon =\varepsilon_{\mathrm{NL}\mathrm{elastic}}\;+\varepsilon_{\mathrm{viscous}},\;\left\{\begin{array}{cc} \sigma & =\mu \varepsilon_{L\mathrm{elastic}}\;+A\varepsilon_{\mathrm{NL}\mathrm{elastic}}^{2}\\ \frac{d\varepsilon_{\mathrm{viscous}}}{dt} & =\frac{\sigma }{\eta } \end{array}\right.$$
  The strain is defined in the models depending on the constitutive assumption considered as, $\varepsilon_{L\mathrm{elastic}}$, $\varepsilon_{\mathrm{NL}\mathrm{elastic}}$ and $\varepsilon_{\mathrm{viscous}}$, or linear elastic strain, nonlinear elastic strain, and viscoelastic strain, respectively. Note that ε is the strain tensor the subscript e.g., Lelastic is referred to linear, nonlinear, or viscoelastic part, respectively. This consideration is defined in the constitutive equation that is useful to establish the different model hypothesis.
$H_{2}$: Maxwell linear viscoelastic, with parameters constants M={μ,η},
  (18) $$\varepsilon =\varepsilon_{L\mathrm{elastic}}+\varepsilon_{\mathrm{viscous}},\;\left\{\begin{array}{cc} \sigma & =\mu \varepsilon_{L\mathrm{elastic}}\\ \frac{d\varepsilon_{\mathrm{viscous}}}{dt} & =\frac{\sigma }{\eta } \end{array}\right.\;\Rightarrow \;\frac{d\varepsilon }{dt}=\frac{\sigma }{\eta }+\frac{1}{\mu }\frac{d\sigma }{dt}$$
$H_{3}$: Linear elastic, with parameters constants M={μ},
  (19) σ=με
$H_{4}$: Maxwell viscoelasticity with third order nonlinear elasticity, parameters constants M={μ,η,A,D} governed by,
  (20) $$\varepsilon =\varepsilon_{\mathrm{NL}\mathrm{elastic}}+\varepsilon_{\mathrm{viscous}},\;\left\{\begin{array}{cc} \sigma & \;=\mu \varepsilon_{L\mathrm{elastic}}+A\varepsilon_{\mathrm{NL}\mathrm{elastic}}^{2}+D\varepsilon_{\mathrm{NL}\mathrm{elastic}}^{3}\\ \frac{d\varepsilon_{\mathrm{viscous}}}{dt} & =\frac{\sigma }{\eta } \end{array}\right.$$
$H_{5}$: model $H_{1}$ combined with a second phenomenological model that states that η=3μ·t as dynamic viscosity where t is time in seconds.

**Figure 3:**
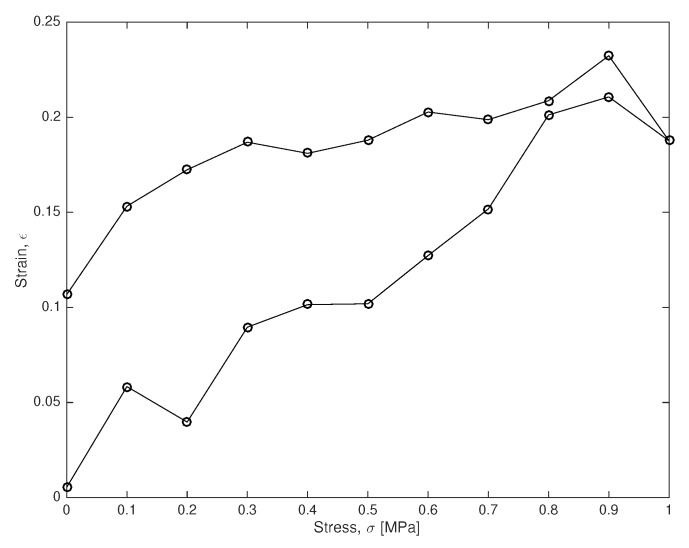
Example of stress control test. Each circle stands for a recorded measurement. The lower branch is the loading curve, whereas the upper one is the unloading curve.

The test is defined as a stress-controlled loading and unloading test at constant velocity between 0 and 1 [MPa] and duration 2T=2 [s]. It yields the simulated stress-strain curve in Figure 3, where measurements are taken every 0.1 s.

To validate the capability of the method under fully controlled uncertainties, instead of real data, the experiment was simulated from model $H_{1}$ with μ=1 [MPa], η=10 [MPa·s] and A=15 [kPa], by adding Gaussian noise simulated with a significative standard deviation of 10 [kPa].

The probabilistic inversion is carried out by joining the experimental information from the experimental stress-strain curves $O=\{\varepsilon_{i}^{o}\}$ with the models above.

### 4.1. Model Parameters Reconstruction

**Figure 4:**
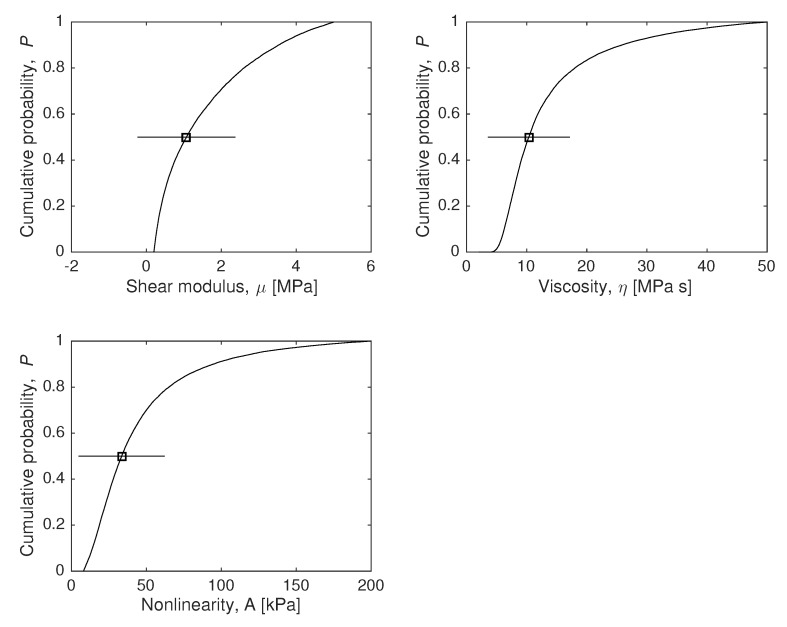
Marginal plausibility maps of model parameters using the model $H_{1}$. The expected values (*p* = 50% value) are marked, as well as their standard deviation bars.

To answer the question of how much we can know about the values of the constitutive constants M={μ,η,A}, under hypothesis $H_{1}$, the marginal probability density can be computed using Equations (10) (a continuous formulation is introduced using integrals) and (21). because in this example, the time dimension is discretized, which forces the use of Monte-Carlo approximation. The results are shown in Figure 4.

The integral in Equation (11) is approximated computationally by a standard Monte Carlo sampling, which approximates the integral of any integrand f(x) that depends on the parameters *x* over a parameter subspace Ω using,

(21) $$\int_{\Omega }f\left(x\right)=\frac{1}{N}\sum_{n=1}^{N}f\left(x_{i}\right)$$

where the integrand f(x) is evaluated at *N* random points $x_{i}\in \Omega$ called samples. The precision is controlled by the number of samples, here chosen as $N=2^{16}$ points, which takes a few seconds on a laptop. Note that, in each hypothesis, the physical parameters that are not present in the formulation are not assigned value zero, but rather not assigned any preferential information density. In other words, this is equivalent to assigning the noninformative (constant) information density over the non-used models’ parameters. When numerically solving the problem, such parameters are actually never assigned any value. The integral is only performed at the computation of the model-class selection.

Considering that we only have 21 experimental data $\varepsilon_{i}^{o}$ (21 circles in Figure 3) with a significative simulated measurement error (10% Gaussian noise on each data), all parameters are successfully estimated (squares with error bars on each plot of Figure 4), as well as their certainty and the shape of the distribution function.

**Figure 5:**
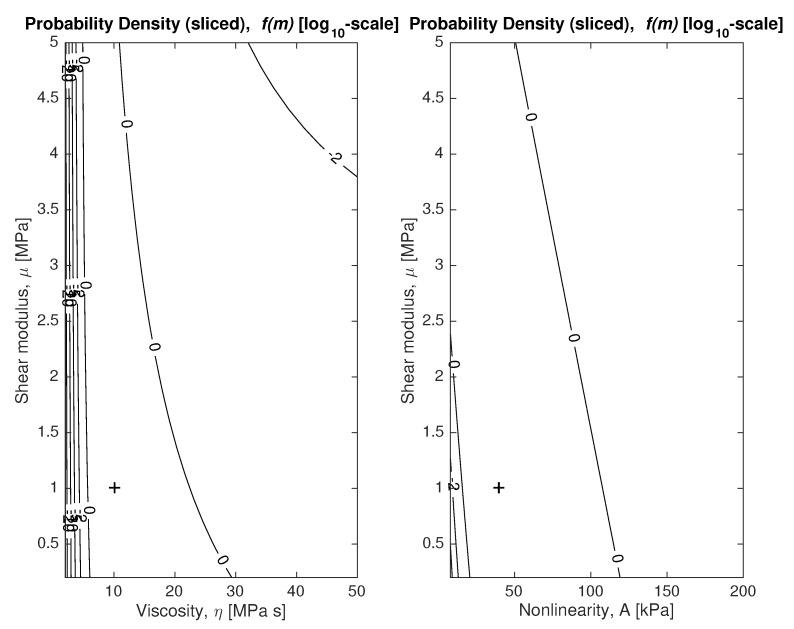
Plausibility maps of model parameters {μ,η} (**left**) and {μ,A} (**right**) given hypothesis $H_{1}$. Plus sign represents optimal shear modulus and viscosity (**left**) and shear modulus and Nonlinear Elastic Constant A (**right**).

To answer the question of how coupled or entangled the unknown model parameters M={μ,η,A} are, visualizing the plausibility maps, which is a $R^{3}\to R$ function, would require a 4-dimensional plot. Instead, we slice it in two 2D contour plots. The slices mean that the model parameters M={μ,η,A} are evaluated by moving two and by fixing the remaining parameters at the most probable values. The contour plots are shown in Figure 5, where the optimal viscosity parameter η is 10 [MPa · s] and it is marked with a plus sign in the plot.

Note that the figure on the right reveals a strong correlation between the linear and nonlinear shear moduli (usually considered in biomechanical characterization [38]), which is a factor for the ill-conditioning of the inverse problem.

### 4.2. Model Hypothesis Ranking

**Figure 6:**
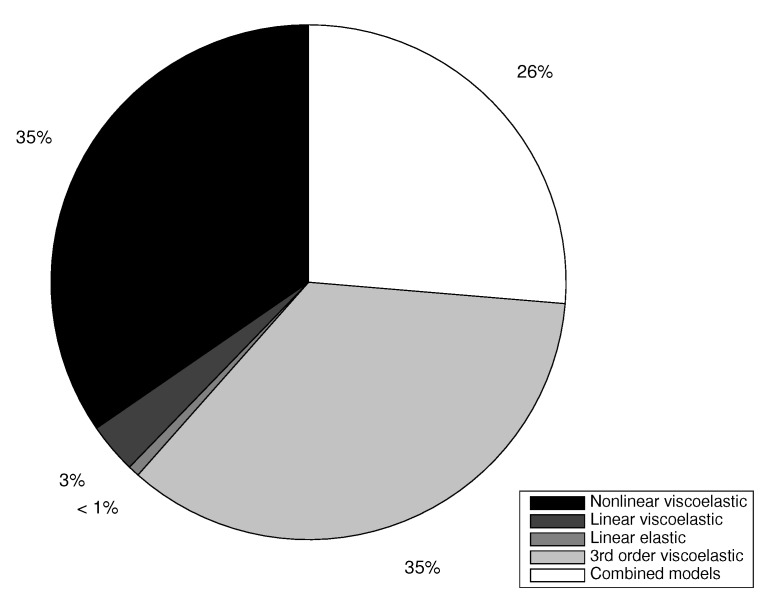
Ranking of model hypothesis.

To answer the question of how much we can trust the assumed physics among a set of candidates, or which model complexity is best by assuming known or unknown physical constants, the model hypothesis raking of the five hypothesis described above is computed using Equations (13) and (21) in Figure 6.

For clarity, the degrees of hypothesis reliability is presented in % by rescaling the information density from Equation (13) as,

(22) $$p\left(H=H_{k}\right)=k_{8}f\left(H=H_{k}\right){|}_{S=S_{l}}=k_{9}\int_{M}e^{-J(M,H_{k},S_{l})}dM\;|\;k_{9}=\frac{1}{\sum_{k}\int_{M}e^{-J(M,H_{k},S_{l})}dM}$$

**Figure 7:**
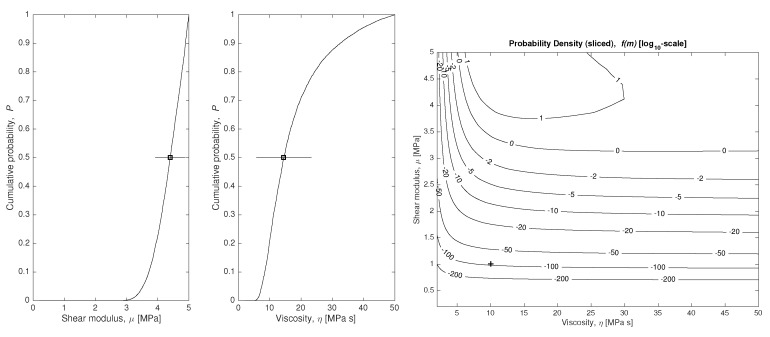
Left: marginal plausibility maps of model parameters using the model $H_{2}$. The expected values (*p* = 50% value) are marked, as well as their standard deviation bars. Right: plausibility maps of the model parameters using the model $H_{2}$.

As an example, the reconstruction of the nonlinear experimental data using the model $H_{2}$ and the corresponding plausibility map contour plots are shown in Figure 7,

Note that the reconstructed parameters are distant from the ones used for the simulation, which is to be expected. The badness of the fitting is also quantified by its low plausibility shown in the ranking in Figure 6.

### 4.3. Interrogation System Optimization

**Figure 8:**
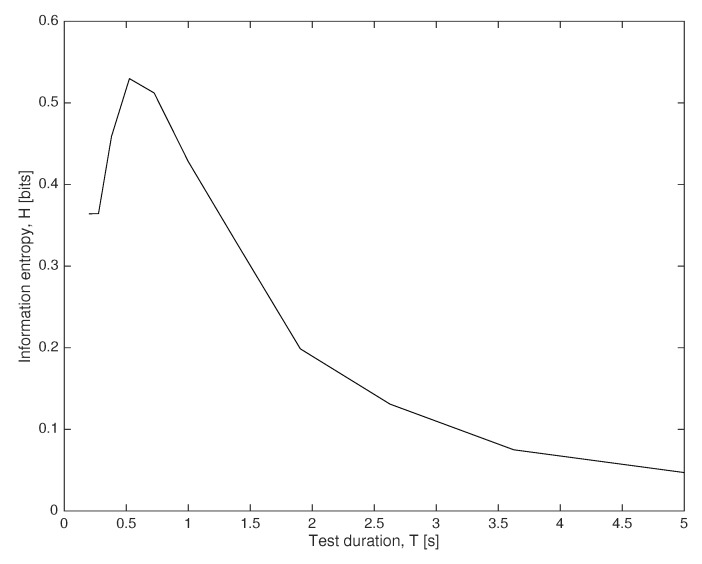
Interrogation system optimization.

Finally, the problem of sensor optimization is illustrated by solving the optimal testing duration S={T}, within a search range T∈[0.2,5] s. Despite the CPU time for solving this low-dimensional problem is quite small, in large-dimensional problems the computational time is expected to be large, and scalability should be studied carefully. The entropy *H* is computed using Equations (16) and (21) and yields the optimal testing configuration using duration T=0.5 [s], as shown in Figure 8 sensor information gain dependence on its design.

The case where some models are particular cases of others (for instance $H_{2}$ is $H_{1}$ with A=0, or $H_{3}$ is $H_{2}$ with η=∞), will not yield the same plausibility, nor zero plausibility, contrarily to first intuition. Note the model complexity is automatically penalized as the integral in Equation (13). It is performed over a higher dimensionality since the model space has as many dimensions as parameters, yielding smaller integrands. As Beck discusses [9,37], this is a mathematical version of Occam’s razor, which prefers the simplest yet accurate model to observations.

## 5. Conclusions

This work presents a new framework to solve probabilistic inverse problems. The framework inherits the ability to move away from the causality relationships of the Bayesian inference formalism from Tarantola [7,14]. Dropping the Cox’s normalization was also done previously [21] exceeding the limits of causality relationships and allowing for a straightforward formulation and computation of realistic problems. This includes multiple concurrent models and stochastic associations, by means of an information-theoretic framework to merge information sources, and a measure of information density that drops Cox’s normalization in favor of strong simplifications, which allows useful generalizations.

These simplifications that arise with the purpose of avoiding the extensive denominator that appears in Bayes’ theorem when the parameters of the model are reconstructed. This metric just requires that the parameters be of Jeffrey’s type, which is usually achievable just through a logarithmic mapping, and is used with the concept of combining information density functions from two independent sources: (i) experimental measurements and (ii) mathematical models, over the observations and model parameters, with the aim of finding which ones are all plausible at the same time.

The derived formulation, beyond the typical estimate of model parameters in a probabilistic way (which answers the question of how much can we know about their values), simplifies a straightforward extension that delivers: (i) the sensor information gain depending on its design, which provides a simpler approach to optimize the experimental design, sensor design or placement; (ii) the plausibility of alternative model hypothesis (which answers the question of how much we can trust the assumed physics among a set of candidates, or which model complexity is best by dropping parameters); and (iii) facilitates multiple concurrent models, which provides practical solutions where multimodal, multiscale or multiphysics are relevant. In addition to this, the framework overrides Bayes’ theorem’s requirement of a causal model, paving the way to contingent models such as stochastic associations, for a start. Further extensions as regularization problems under this approach will be considered in the future and could also be easily extracted, such as robust model reconstruction, or adding new dimensions to the problem to accommodate future real-world needs.
